# The significance of microRNA-148/152 family as a prognostic factor in multiple human malignancies: a meta-analysis

**DOI:** 10.18632/oncotarget.17949

**Published:** 2017-05-17

**Authors:** Chenkui Miao, Jianzhong Zhang, Kai Zhao, Chao Liang, Aiming Xu, Jundong Zhu, Yuhao Wang, Yibo Hua, Ye Tian, Shouyong Liu, Chao Zhang, Chao Qin, Zengjun Wang

**Affiliations:** ^1^ State Key Laboratory of Reproductive Medicine and Department of Urology, The First Affiliated Hospital of Nanjing Medical University, Nanjing, China

**Keywords:** miR-148/152 family, human malignancies, prognosis, meta-analysis

## Abstract

Recent studies have demonstrated that microRNA-148/152 family emerges as a attractive biomarker for predicting tumor prognosis and progression. However, outcomes of different studies are controversial. Eligible Literature were searched through online databases: PubMed, EMBASE and Web of Science. A total of 24 eligible studies were ultimately enrolled in this meta-analysis. Results indicated that overexpression of miR-148/152 family was significantly correlated with enhanced overall/cause-specific survival (OS/CSS) (HR=0.63, 95% CI: 0.54-0.74). Stratified analysis indicated that high miR-148a and miR-148b expression predicted favorable OS/CSS (HR=0.76; 95% CI: 0.69-0.90) and (HR=0.49; 95% CI: 0.39-0.61), while miR-152 developed no significant impact (HR=0.40, 95% CI: 0.12-1.29). MiR-148/152 family was distinctly associated with superior OS/CSS in Asian (HR=0.53, 95% CI: 0.44-0.64), but not in Caucasian (HR=0.96, 95% CI: 0.82-1.13). Futhermore, miR-148/152 family expression also predicted longer disease/relapse/progression-free survival (DFS/RFS/PFS) (HR=0.37, 95% CI: 0.16-0.88). A significantly favorable DFS/RFS/PFS was observed in Asian (HR=0.21, 95% CI: 0.06-0.81) than that in Caucasian (HR=0.76, 95% CI: 0.31-1.87). miR-148/152 family overexpression also predicted longer DFS/RFS/PFS in tissues (HR=0.11, 95% CI: 0.01-0.98), but not in plasma/serum (HR=0.67, 95% CI: 0.38-1.18). Our meta-analysis demonstrated that overexpression of miR-148/152 predicted enhanced OS/CSS and DFS/RFS/PFS of cancer patients. MiR-148a/b family may serve as a potential prognostic factor in multiple human malignancies.

## INTRODUCTION

MicroRNAs (miRNAs) are class of non-coding small RNAs which approximately range from 18-25 nucleotides in length. Mature miRNAs regulate a wide variety of target genes in post-transcriptional level by binding to the 3′-untranslated complementary sequence of messenger RNA (mRNA) [1, 2]. Thus, miRNAs play pivotal roles in gene expression and diverse biological processes, such as cell proliferation, cycle, apoptosis and differentiation [3–5]. Emerging studies have declared that miRNAs were proposed as predictive indicators for multiple human neoplasms due to the aberrant expression discrepancy between tumor tissues and normal tissues [6–8]. In 2002 Calin et al first reported the biological role of miR-15 and miR-16 in chronic lymphocytic leukemia, this was the first time to investigate the relationship between microRNA and cancer [9]. Additionally, miRNAs are generally classified into two categories: hazardous miRNAs which correlated with poor prognosis and protective miRNAs which are known as favorable survival predictors [10–12]. As a member of miRNAs, miR-148/152 family have been reported to develop prognostic role in multiple carcinomas.

The miR-148/152 family consists of three highly homologous members (miR-148a, miR-148b, and miR-152), of which ectopic expression was observed in multiple diseases such as: atherosclerosis, diabetes, and cancers [13–15]. The majority of studies have considered miR-148/152 family as a tumor suppressor and exerted anti-tumor effect in human neoplasms [16–18]. For instance, Qiu et al reported that miR-148a expression was down-regulated in gastric tumor tissues compared with non-tumor tissues. Evaluated expression of miR-148a significantly predicted favorable overall survival of patients with gastric cancer [19]. Ma et al detected the decreased miR-148a level in bladder carcinoma specimens and reduced miR-148a expression correlated with shorter survival time and increased recurrence risk [20]. In addition, downregulation of miR-148b was found to associate with poorer outcomes in patients with hepatocellular carcinoma [21], and high miR-152 expression developed a negative impact on recrudescence in NSCLC [22]. However, there still exists a series of investigations presenting an adverse function of miR-148/152 family, indicating that the correlation between them remains controversial. Kjersem et al have declared that evaluated miR-148a predicted pernicious progression and shoter overall survival of colorectal cancer patients [23]. Furthermore, Wang et al reported the onceogenic value of miR-152 in colorectal carcinoma, but failed to demonstrate a significant impact on prognosis [24]. Therefore, consensus has not been reached to the reliability of miR-148/152 family as prognostic indicators in various human neoplasms.

Considering the limitation of study scale, we sought to carry out this meta-analysis to summarize available findings and clarify the predictive significance of miR-148/152 family in malignancies prognoses.

## RESULTS

### Overview of eligible studies

**Figure 1:**
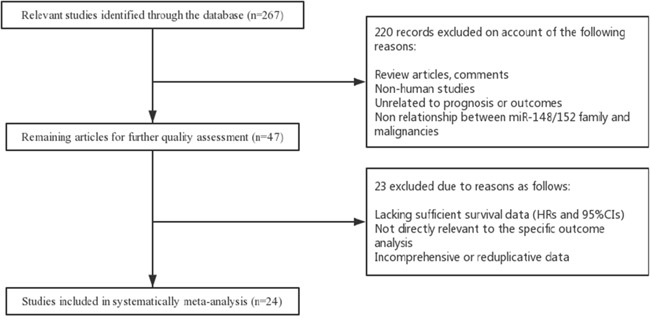
Flow diagram of literature search and selection process

A total of 267 studies from published database PubMed, EMBASE, and the Web of Science were identified to focus on the association between miR-148/152 family expression and multiple human malignancies. After a manual screening of titles and abstracts, 220 studies were excluded on account of the following reasons: review articles or letters, not human studies, unrelated to prognosis or outcomes, no relationship between miR-148/152 family and malignancies. For further quality evaluation of remaining candidates, 23 potential studies were excluded due to insufficient survival data, indirectly related to specific prognosis, incomprehensive or reduplicative data. Finally, 24 studies were considered to be included in the meta-analysis. The selection process of candidate studies are presented in detail in Figure 1.

Dominant characteristics of included investigations were summarized in Table 1 and Table 2. For data extracted from the 24 eligible studies, 13 evaluated the relationship between miR-148a and human carcinomas, while 9 reported miR-148b and other 5 focused on miR-152. For survival analyses, 25 focused on patients OS/CSS and 8 reported DFS/RFS/PFS. Of these eligible studies, 20 evaluated Asian population while other 4 studies concentrated on Caucasian. In addition, the malignant neoplasms consisted of hepatocellular carcinoma (HCC), non-small cell lung cancer (NSCLC), colorectal cancer (CRC), and bladder cancer, ovarian cancer, gastric cancer and pancreatic cancer, skin cancer, endometrial serous adenocarcinoma and Osteosarcoma. All of the analyzed studies were retrospective except for one prospective. 14 studies focused on pathological type of adenocarcinoma (AdenoCA), 2 evaluated squamous carcinoma (SqCa), 3 reported (SqCa/AdenoCA) and 2 assessed AdenoCA/Mucinous, other 3 studies focused on epithelial carcinoma, transitional cell carcinoma and sarcoma, respectively. Quantitative real-time PCR (qRT-PCR) was widely used in all eligible studies to calculate miRNA-148/152 family expression.

**Table 1:** Main characteristics of eligible studies in the meta-analysis

First author, publication year	MicroRNA type	Case nationality	Median or mean age	Dominant ethnicity	Study design	Malignant disease	Main type of pathology	Detected sample	Survival analysis	Source of HR	Maximum months of follow-up
Qiu,2016	miR-148a	China	60	Asian	R	Gastric cancer	AdenoCA	Tissue	OS	Reported	60
NG,2016	miR-148a	China	55	Asian	R	HCC	AdenoCA	Plasma	OS/DFS	Reported	138
Wang,2016	miR-152	China	60	Asian	R	CRC	AdenoCA	Tissue	OS	Reported	54
Wang,2016	miR-148b	China	60	Asian	R	NSCLC	SqCa/AdenoCA	Tissue	OS	Reported	60
Gong,2016	miR-148a	China	50	Asian	R	Ovarian cancer	Epithelial carcinoma	Plasma	OS/RFS	Reported	60
Ma,2016	miR-148a	China	60	Asian	R	Bladder cancer	Transitional cell carcinoma	Tissue	OS/RFS	Reported/SC	120
Ziari,2016	miR-148b	Iran	60	Asian	R	HCC	AdenoCA	Tissue	OS	Reported	80
Wang,2016	miR-148a	China	51	Asian	R	HCC	AdenoCA	Serum	OS	Reported	35
Wang,2016	miR-148b	China	51	Asian	R	HCC	AdenoCA	Serum	OS	Reported	35
Wang,2016	miR-152	China	51	Asian	R	HCC	AdenoCA	Serum	OS	Reported	35
Tian,2015	miR-148a	China	60	Asian	R	Skin cancer	SqCa	Tissue	OS	Reported	60
Wang,2015	miR-152	China	19	Asian	P	Osteosarcoma	Sarcoma	Tissue	OS	Reported	60
Hibino,2015	miR-148a	Japan	63	Asian	R	CRC	AdenoCA	Tissue	CSS	Reported	60
Ghasemkhani, 2015	miR-148b	Iran	50	Asian	R	NSCLC	SqCa/AdenoCA	Tissue	OS	Reported	60
Sadeghian,2015	miR-148b	Iran	60	Asian	R	HCC	AdenoCA	Tissue	OS	Reported	60
Ge,2015	miR-148b	China	60	Asian	R	NSCLC	SqCa/AdenoCA	Tissue	OS	Reported	60
Zhang,2015	miR-148b	China	50	Asian	R	HCC	AdenoCA	Tissue	OS	SC	48
Zhang,2014	miR-148b	China	50	Asian	R	HCC	AdenoCA	Tissue	OS	Reported	80
Sakamoto,2014	miR-148a	Japan	60	Asian	R	Gastric cancer	AdenoCA	Tissue	OS	Reported	66
Kjersem,2014	miR-148a	Norway	60	Caucasian	R	CRC	AdenoCA	Plasma	OS/PFS	Reported	NM
Sanfiorenzo, 2013	miR-152	France	65.1	Caucasian	R	NSCLC	SqCa	Plasma	DFS	Reported	NM/SC
Tsai,2013	miR-148a	China	65	Asian	R	CRC	AdenoCA/Mucinous	Serum	OS/DFS	SC	67
Christensen,2013	miR-148a	Denmark	66	Caucasian	R	CRC	AdenoCA/Mucinous	Tissue	DFS	SC	96
Zhao,2013	miR-148b	China	60	Asian	R	Pancreatic Cancer	AdenoCA	Tissue	OS	SC	40
Schultz,2012	miR-148a	Denmark	64	Caucasian	R	PDAC	AdenoCA	Tissue	OS	Reported	300
Schultz,2012	miR-148a	Denmark	64	Caucasian	R	A-AC	AdenoCA	Tissue	OS	Reported	300
Hiroki,2009	miR-152	Japan	64.9	Asian	R	Endometrial serous adenocarcinoma	AdenoCA	Tissue	OS/DFS	Reported	64

Study design is described as prospective (P) or retrospective (R). miR-148a, microRNA-148a; miR-148b, microRNA-148b; miR-152, microRNA-152.

AdenoCA, adenocarcinoma; SqCa, squamous carcinoma; OS, overall survival; CSS, cause-specific survival; DFS, disease-free survival; RFS, recurrence-free survival; PFS, progression-free survival.

HCC, hepatocellular carcinoma; NSCLC, non-small cell lung cancer; CRC, colorectal cancer; PDAC, pancreatic ductal adenocarcinoma; A-AC, ampullary adenocarcinomas.

NM, not mentioned; SC, survival curve.

**Table 2:** HRs and 95% CIs of patient survival or cancer progression relating to MMPs expression in eligible studies

First author, publication year	MicroRNA type	Main assay method	Cut-off value	Case number		OS/CSS		DFS/RFS/PFS	
				High expression	Low expression	HR(95%CI)(U/M)	P Value	HR(95%CI)(U/M)	P Value
Qiu,2016	miR-148a	qRT-PCR	median	39	55	0.80(0.65-0.99)U	0.045	NM	NM
NG,2016	miR-148a	qRT-PCR	NM	31	31	0.45(0.03-6.25)M	0.548	0.43 (0.02-7.57)M	0.561
Wang,2016	miR-152	qRT-PCR	median	101	101	1.171(0.479-2.864)M	0.73	NM	NM
Wang,2016	miR-148b	qRT-PCR	NM	NM	NM	0.48(0.32-0.93)M	0.003	NM	NM
Gong,2016	miR-148a	qRT-PCR	median	55	47	0.589(0.41-0.85) M	0.005	0.70 (0.49-1.O1)M	0.058
Ma,2016	miR-148a	qRT-PCR	median	56	70	0.60(0.38-0.96)U*	0.005	0.07 (0.045-0.148)M	<0.001
Ziari,2016	miR-148b	qRT-PCR	median	NM	NM	0.452(0.110-0.699)M	0.012	NM	NM
Wang,2016	miR-148a	qRT-PCR	median	38	38	0.442(0.212-0.923)M	0.03	NM	NM
Wang,2016	miR-148b	qRT-PCR	median	38	38	0.709(0.343-1.462)M	0.352	NM	NM
Wang,2016	miR-152	qRT-PCR	median	38	38	0.578(0.278-1.2)M	0.141	NM	NM
Tian,2015	miR-148a	qRT-PCR	NM	50	55	0.053(0.005-0.548)M	0.014	NM	NM
Wang,2015	miR-152	qRT-PCR	mean	38	42	0.126(0.023-0.7010)M	0.004	NM	NM
Hibino,2015	miR-148a	qRT-PCR	median	16	33	0.226(0.054-0.679)M	0.006	NM	NM
Ghasemkhani, 2015	miR-148b	qRT-PCR	median	58	46	0.32(0.09-0.65)M	0.021	NM	NM
Sadeghian, 2015	miR-148b	qRT-PCR	median	NM	NM	0.378(0.19-0.57)M	0.01	NM	NM
Ge,2015	miR-148b	qRT-PCR	median	74	77	0.424(0.109-0.62)M	0.011	NM	NM
Zhang,2015	miR-148b	qRT-PCR	median	20	20	0.64(0.16-2.56)U*	<0.01	NM	NM
Zhang,2014	miR-148b	qRT-PCR	median	58	98	0.54(0.34-0.81)M	0.002	NM	NM
Sakamoto,2014	miR-148a	qRT-PCR	median	50	52	0.325(0.114-0.822)M	0.0169	NM	NM
Kjersem,2014	miR-148a	qRT-PCR	median	NM	NM	1.179(0.96-1.45)M	0.118	1.290 (1.07-1.55)M	0.007
Sanfiorenzo, 2013	miR-152	qRT-PCR	mean	NM	NM	NM	NM	0.333 (0.125-0.892)M	0.029
Tsai,2013	miR-148a	qRT-PCR	mean	55	55	0.58(0.29-1.17)U*	0.0156	0.43(0.20-0.90)U*	0.0006
Christensen, 2013	miR-148a	qRT-PCR	median	20	26	NM	NM	0.78 (0.22-2.76)U*	0.0236
Zhao,2013	miR-148b	qRT-PCR	median	24	24	0.56(0.25-1.25)U*	<0.05	NM	NM
Schultz,2012	miR-148a	qRT-PCR	median	NM	NM	0.97(0.91-1.04)M	0.54	NM	NM
Schultz,2012	miR-148a	qRT-PCR	median	NM	NM	0.82(0.74-0.91)M	<0.001	NM	NM
Hiroki,2009	miR-152	qRT-PCR	median	NM	NM	0.005(4.77E-5-0.440)M	0.021	0.003 (4.37E-5-0.250)M	0.01

The source of HR and 95% CI was extracted from survival curves or article reports. miR-148a, microRNA-148a; miR-148b, microRNA-148b; miR-152, microRNA-152.

*HR calculated from survival curves; SC, survival curve; U, univariate analysis; M, multivariate analysis; NM, not mentioned.

OS, overall survival; CSS, cause-specific survival; DFS, disease-free survival; RFS, recurrence-free survival; PFS, progression-free survival.

qRT-qPCR, reverse transcriptase-quantitative PCR.

### Patients survival associated with miR-148/152 expression

**Figure 2:**
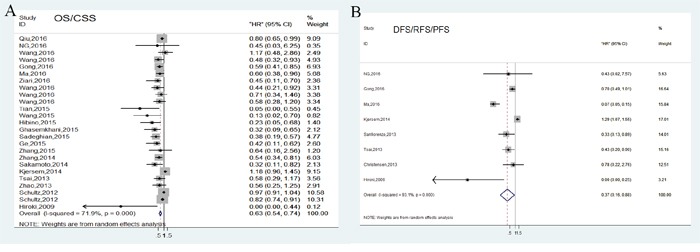
Forest plots of pooled analyses associated with miR-148/152 family expression Survival data are reported as overall survival/cause-disease survival (OS/CSS) **(A)** and disease-free survival/recurrence-free survival/progression-free survival (DFS/RFS/PFS) **(B)**.

**Figure 3:**
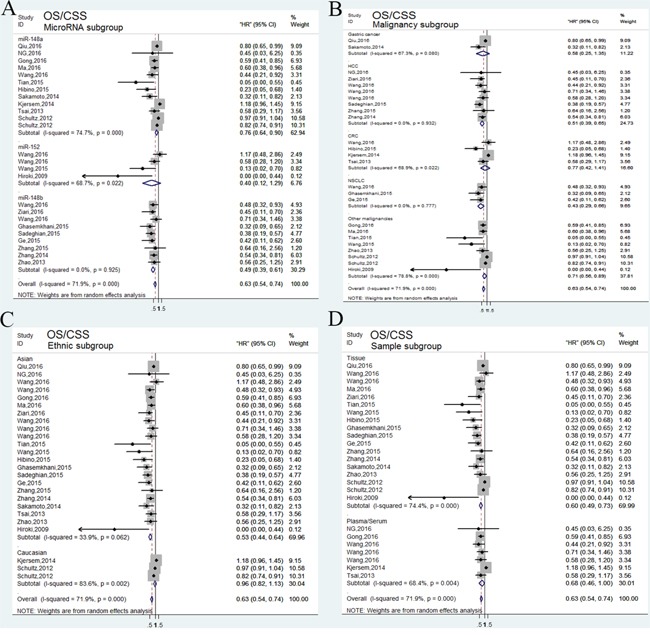
Forest plots of subgroup analysis of the OS/CSS **(A)** stratified by MicroRNA subgroup; **(B)** stratified by malignancy subgroup; **(C)** stratified by ethnic subgroup; **(D)** stratified by sample subgroup.

For studies evaluating OS/CSS analysis, a random-effects model was performed due to significant heterogeneity (*P*<0.001, *I^2^*=71.9%). Our analyses indicated that high expression of miR-148/152 family could significantly predict a favorable OS/CSS for various human carcinomas, with a combined HR of 0.63 (95% CI: 0.54-0.74, Figure 2A). Furthermore, we carried out stratified analyses by classifying studies into subgroups. Results from subgroups suggested that miR-148a and miR-148b exerted enhanced OS/CSS, with a pooled HR of 0.76 (95% CI: 0.69-0.90) and 0.49 (95% CI: 0.39-0.61), while abnormal miR-152 expression developed no statistical impact (HR=0.40, 95% CI: 0.12-1.29; Figure 3A). In stratified analyses with cancer types, 6 studies reporting HCC and 3 reporting NSCLC indicated that miR-148/152 family were particularly associated with favorable OS/CSS (HCC: HR=0.5, 95% CI: 0.39-0.65; NSCLC: HR=0.43, 95% CI: 0.29-0.66; Figure 3B). Other 4 studies demonstrated that miR-148/152 family exerted no significant function on OS/CSS in CRC patients (HR=0.77, 95% CI: 0.42-1.41; Figure 3B), and 2 studies with gastric cancer obtained a similar result (HR=0.58, 95% CI: 0.25-1.35; Figure 3B). In addition, up-regulated miR-148/152 family correlated with superior OS/CSS in Asian (HR=0.53, 95% CI: 0.44-0.64) than that in Caucasian population (HR=0.96, 95% CI: 0.82-1.13; Figure 3C). Considering the limitation of investigation quantity, the prognostic significance of miR-148/152 family in other caner types still needed further confirmation.

### Tumor progression associated with miR-148/152 expression

**Figure 4:**
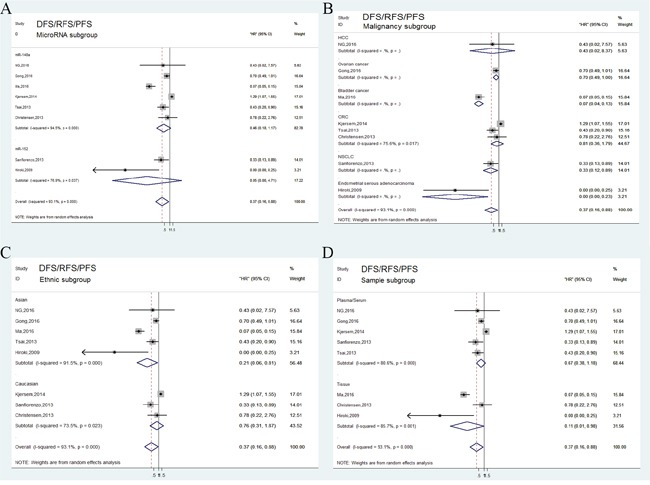
Forest plots of subgroup analysis of the DFS/RFS/PFS **(A)** stratified by MicroRNA subgroup; **(B)** stratified by malignancy subgroup; **(C)** stratified by ethnic subgroup; **(D)** stratified by sample subgroup.

In general, tumor progression was assessed by combining disease recurrence and metastasis. A total of 8 independent studies reported DFS/RFS/PFS analysis and revealed a protective significance of upregulated miR-148/152 family expression in multiple human neoplasms (HR=0.37, 95% CI: 0.16-0.88; Figure 2B). A random-effects model was applied to estimation due to a significant heterogeneity between studies (*P*<0.001, *I^2^*=93.1%). Stratified analyses indicated that miR-148/152 overexpression was a significant prediction for tumor recurrence and progression in tissues (HR=0.11, 95% CI: 0.01-0.98) but not in plasma/serum (HR=0.67, 95% CI: 0.38-1.18; Figure 4D). In ethnic subgroups, our analysis suggested that high miR148/152 expression correlated with favorable DFS/RFS/PFS in Asian population (HR=0.21, 95% CI: 0.06-0.81), but failed to obtain a significant consequence in Caucasian (HR=0.76, 95% CI: 0.31-1.87; Figure 4C). Results from other stratified analysis were presented in Figure 4A and 4B.

### Sensitivity analyses

**Figure 5:**
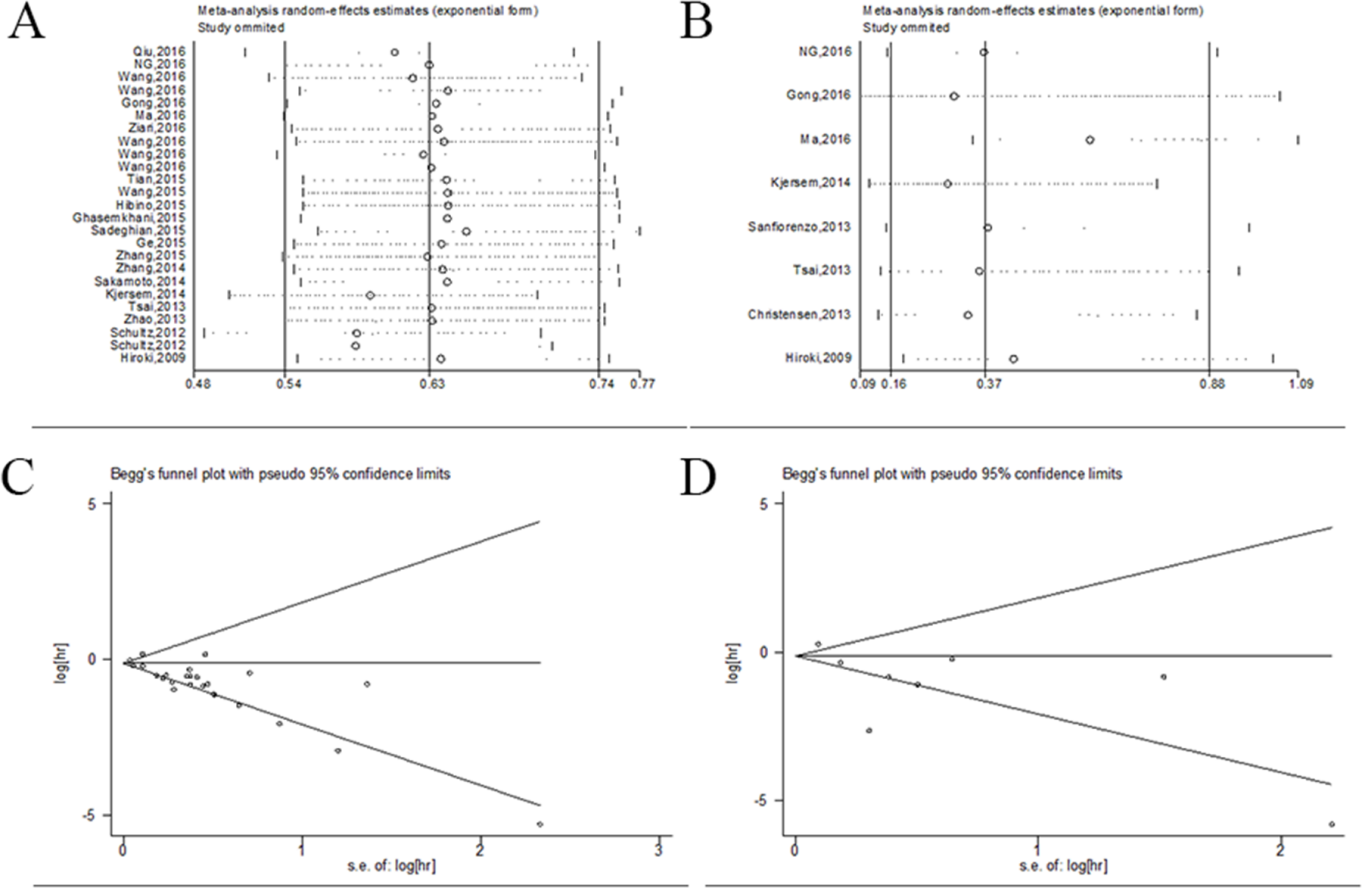
Sensitivity and bias analysis under specific model **(A)** effect of individual studies on the pooled HR for OS/CSS; **(B)** effect of individual studies on the pooled HR for DFS/RFS/PFS; **(C)** Begg's funnel plots of publication bias for OS/CSS. **(D)** Begg's funnel plots of publication bias for DFS/RFS/PFS.

We also conducted sensitivity analyses by sequentially omitting individual studies to evaluate whether exclusion of any individual study indicated alterations in the results. The sensitive analyses from a random-effect model indicated the analyzed results was stable (Figure 5A, 5B).

### Publication bias

Publication bias of the included investigations was performed by funnel plots and Begg's tests. In the combined prognostic analysis as determined, the funnel plots were symmetric and *P* values were 0.118 for OS/CSS and 0.536 for DFS/RFS/PFS (*P*>0.05), respectively (Figure 5C, 5D). Therefore, no significant publication bias was observed in the meta-analysis.

## DISCUSSION

Recently, exhaustive efforts have been invested in identifying prognostic biomarkers for patients with multiple malignancies. Mounting evidence has indicated that microRNAs play crucial roles in carcinogenesis and cancer progression, which are closely associated with various biological activities such as cell proliferation, cycle, invasion, and metastasis [25]. MicroRNAs are more stably expressed in multiple specimen samples compared with mRNAs and proteins, which can be accurately quantified by qRT-PCR [26, 27]. Therefore, an increasing number of investigations have verified microRNAs as potential targets for clinical treatment, as well as promising biomarkers for cancer prognoses [28–30].

Currently, miR-148/152 family members include miR-148a, miR-148b, and miR-152, of which the three share a common seeds sequence in domains [31]. Previous studies have confirmed that down-regulation of miR-148/152 family is associated with unfavorable survival and prognostic outcomes of patients with malignancies [32–34]. Downregulated miR-148a expression was found in gastric tumor compared with non-neoplastic mucosa, and this was correlated with advanced tumor invasiveness and poor prognosis by targeting MMP7 [35]. Zhang et al found that decreased miR-148b expression in HCC predicted poor prognosis, and downregulated miR-148b significantly enhanced cancer progression with advanced vein invasion and TNM stage [36]. In addition, Wang et al detected the circulating miR-148a, miR-148b, and miR-152 and revealed that loss of miR-148a expression independently predicted a shorter overall time in patients with HCC than miR-148b and miR-152 [37]. Even though, certain investigations have presented contradictory results. For instance, loss level of miR-148 was found to predict longer period of recurrence and favorable overall survival in esophageal adenocarcinoma patients [38]. Ma et al confirmed that miR-148a high expression was an independent indicator for unfavorable overall survival and disease-specific survival, respectively [39]. The observation of these oncogenic role of miR-148/152 family in multiple cancers might cast doubt on its dominant anti-tumor effects. Despite these controversial results, miR-148/152 family was still an attractive biomarker for considerable prognostic significance.

The prognostic role of miR-148/152 family in human neoplasms may partly attributed to its underlying molecular mechanism, as well as dissimilar biological function. In HCC patients, miR-148a overexpression was found to suppress cell invasion and affects prognosis by directly targeting sphingosine-1-phosphate receptor 1(S1PR1) [40, 41]. He et al verified that miR-148a inhibited NSCLC cell proliferation and invasion activity through silencing signal transducer and activator of transcription 3 (STAT3), which highlighted miR-148a/STAT3 axis as a potential target for clinical treatment with NSCLC patients [42]. Kim et al also found that miR-148a acted as a tumor suppressor and holds vital potential for renal carcinoma therapy by directly targeting Rab14 [43]. Furthermore, miR-148/152 family was found to involved into DNA methylation by interacting with DNA methyltransferase enzyme 1 (DNMT1) in many malignancies types. Zhu et al found that DNMT1 overexpression inactivated miR-148a by hypermethylation of DNA in gastric cancer. Inhibition of miR-148a might promote DNA hypermethylation in case of the overexpression of DNMT1 [44]. In a breast cancer study, Xu et al that high DNMT1 expression was responsible for hypermethylation of miR-148a and miR-152 promoters. Besides, DNMT1 was conversely associated with miR-148a/152 expression, which highlighted a potential miR-148a/152-DNMT1 regulatory framework might exist in breast cancer [45]. Based on these underlying mechanism, we concluded that miR-148/152 family in specific cancer category might induce particular biological behaviors.

In this meta-analysis, we first collected available data from published studies to assess the prognostic significance of miR-148/152 family in multiple human malignancies. Subgroup, sensitivity, and heterogeneity analysis were conducted to explore the effects of main characteristics in relevant studies. Results from OS/CSS analysis indicated that up-regulated miR-148/152 family could predict favorable outcomes with a pooled HR of 0.63 (95% CI: 0.54-0.74). Additionally, the pooled outcome in the DFS/RFS/PFS analysis indicated that increased miRNA-148/152 expression is predictive of slower cancer progression (HR=0.37, 95% CI: 0.16-0.88). In stratified analysis, we found that upregulated miR-148/152 family predicted superior OS/CSS in Asian (HR=0.53, 95% CI: 0.44-0.64), but analysis in Caucasian population failed to obtain the significance (HR=0.96, 95% CI: 0.82-1.13). Similar outcomes of DFS/RFS/PFS analysis in ethnic subgroups was observed that aberrant miR-148/152 expression contributed to favorable disease progression in Asian population (HR=0.21, 95% CI: 0.06-0.81), but not in Caucasian (HR=0.76, 95% CI: 0.31-1.87). Previous investigations have confirmed that specifc miRNAs emerged diverse expression levels and develop particular survival impact in multiple ethnic groups [46, 47]. The occurrence of these discrepancies might be caused by the difference in environmental exposures and genetic backgrounds.

Admittedly, miR-148a, miR-148b, and miR-152 are the three members of the miR-148/152 family with the same seed sequence, of which are pivotal for binding to target mRNAs. In analysis of microRNA subgroups, our results demonstrated that miR-148a and miR-148b promoted favorable OS/CSS (HR=0.76, 95% CI: 0.69-0.90) and (HR=0.49, 95% CI: 0.39-0.61), nevertheless abnormal miR-152 expression exerted no statistical significance (HR=0.40, 95% CI: 0.12-1.29). Diverse prognostic values between miR-148a/b and miR-152 may attributed to different domains of the three, even though they possessed the same seed sequence. In addition, the deficiencies of studies focusing on miR-152 and cancer outcomes also accounted to some extent. Furthermore, malignancies species also had a considerable impact on the prognostic role of miR-148/152 family. Seven survival data from OS/CSS analysis indicated that miR-148/152 family play a vital role in overall survival for patients with HCC (HR=0.51, 95% CI: 0.39-0.65), revealing the independent value of miR-148/152 family in HCC [21, 34, 36, 48–50]. Although analyses of other neoplasms also obtained a statistical outcome, these results need to be further confirmed due to the deficiency of studies. Interesting, stratification analysis of different detected specimens suggested that miR-148/152 served as a significant indicator for tumor recurrence and progression in tissues (HR=0.11, 95% CI: 0.01-0.98) other than plasma/serum (HR=0.67, 95% CI: 0.38-1.18). These might origin from the specificity of diverse samples in various neoplasm and limitations of lacking more researches. To summarize, our meta-analysis indicated that detection of abnormal miR-148/152 family levels is of great significance in predicting prognosis of various human malignancies.

What can not be overlooked is the existence of heterogeneity when accounting for the results of this meta-analysis [51]. As is determined by Stata software, heterogeneity and publication bias of eligible subjects might affect the stability of our demonstration. In this meta-analysis, a significant heterogeneity was observed when we carried out OS/CSS analysis of miR-148/152 family, as well as comparison for DFS/RFS/PFS. All of these might weaken the pooled results of meta-analysis and cannot explicitly states the prognostic status of miR-148/152 family. Based on respective results above, we performed stratified analysis to minimize the impact of heterogeneity by classifying studies into subgroups of microRNA types, dominant ethnicity, malignant diseases, detected samples and pathological categories. Partly decreasing heterogeneity was presented in some subgroups, even though they still existed in the results. Furthermore, sensitivity analysis was also carried out to strengthen the conclusion of the meta-analysis. We found that exclusion of individual studies brought about quite tiny change of estimated pooled HRs. No evidence of significant publication bias was noted in this meta-analysis, indicating our analyzed results were credible.

Although elaborate check was conducted along with statistical analysis, our conclusion still needs further refinement for the following accounts. Firstly, all investigations included were published in English except one Chinese article, which might induced English language bias in pooled results [52, 53]. Secondly, the number of eligible studies reporting DFS/RFS/PFS was not sufficiently enough for a comprehensive analysis. Thirdly, a recognized miR-148/152 expression level could hardly to achieve even the majority of articles regarded the median/mean points as the cut-off value. What's more, only Asian and Caucasian population were analyzed in this meta-analysis, which might weaken the meta-analytic worth to some extent. Considering these limitations, the significance of miR-148/152 family as a prognostic indicator in multiple human malignancies might be overestimated. Our results should be interpreted minutely and need further confirmation.

In summary, this meta-analysis demonstrates that miR-148/152 overexpression can significantly predict favorable prognostic outcomes in diverse human neoplasms, particularly in Asian population and tissues specimens. Besides, miR-148a/b are promising biomarkers for predicting patients overall outcomes than miR-152. Taking insufficient evidence into account, in order to get a better evaluation of the prognostic role in patients with malignancies, further large-scale researches and clinical studies are needed for further convince.

## MATERIALS AND METHODS

### Search strategy

We conducted this meta-analysis in accordance with the standard guidelines of the Meta-analysis of Observational Studies in Epidemiology group (MOOSE) [54]. A literature search through online databases such as PubMed, Embase, and Web of Science were performed up to March 2017, using the following keywords (“microRNA-148a” or “miR-148a” or “microRNA-148b” or “miR-148b” or “microRNA-152” or “miR-152”) and (“cancer” or “carcinoma” or “Neoplasm” or “Tumor”) and (“prognostic” or “prognosis” or “survival” or “outcome” or “recurrence” or “relapse”). Eligible studies to be included in this analysis should meet the following criteria: (1) studies exploring various human malignancies; (2) a relationship between miR-148/152 family and cancer prognosis. With the aim to supplement our literature search, reference lists of eligible studies were screened for additional publications.

### Quality assessment

In order to evaluate the quality of all included studies, we used a critical review checklist of the Dutch Cochrane Centre proposed by MOOSE. The key points of the quality assessment included the following: (1) origin of country and definition of study population, (2) clear microRNA subtypes and carcinoma classifications, (3) the study design and cut-off value of miR-148/152 family, (4) detected samples and pathology, (5) description of outcomes and follow-up period of patients. Studies without specifying the points mentioned above were excluded to maintain the quality of the meta-analysis. A flow diagram of the study selection process is presented in Figure 1.

### Data extraction

Two investigators (Chen-kui Miao and Jian-zhong Zhang) independently identified all eligible studies and extracted relevant data to rule out any discrepancy. Following data elements were included and recorded: (1) the first authors’ names, publication year, and nationality of study population, (2) microRNA type, (3) dominant ethnicity and malignant types, (4) detected samples and pathology, (5) main assay method and cut-off definition, (6) following up duration and (7) HRs associated with evaluated miR-148/152 for OS/CSS and DFS/RFS/PFS along with 95% CIs and P values. If HRs and 95% CIs were only available in Kaplan-Meier curves, data were extracted from graphical survival plots using Engauge Digitizer version 4.1 [55, 56].

### Statistical analysis

Cochran's Q-test and Higgins I^2^ statistics (*I^2^*) were carried out to test the heterogeneity of pooled HRs. *P*<0.0*5* was considered statistically significant. The fixed-effects model (Mantel-Haenszel method) or the random-effects model (DerSimonian-Laird method) was performed for analysis according to the heterogeneity of all eligible investigations. If the heterogeneity was considered significantly at *P*<0.05 or the percentage of *I^2^* was greater than 50%, a random-effects model was applied to calculate the pooled HR, otherwise a fixed-effects model was conducted. In addition, we also executed stratified analyses upon similar characteristics to minimize the sources of heterogeneity. Publication bias was assessed by using Begg's test and Egger's test [57, 58]. All above statistical calculations were conducted by using Stata version 12.0 (Stata Corporation, College Station, TX, USA).
